# Early Clinical Features of Pseudoexfoliation Syndrome in Anterior Segment and Gonioscopy Examination

**DOI:** 10.4274/tjo.62444

**Published:** 2017-01-17

**Authors:** Sirel Gür Güngör, Atilla Bayer, Ahmet Akman, Leyla Asena

**Affiliations:** 1 Başkent University Faculty of Medicine, Department of Ophthalmology, Ankara, Turkey; 2 Dünyagöz Hospital, Ophthalmology Clinic, Ankara, Turkey

**Keywords:** Anterior segment, gonioscopy, pseudoexfoliation syndrome

## Abstract

**Objectives:**

To determine the early signs of pseudoexfoliation (PEX) in fellow eyes of cases with unilateral PEX.

**Materials and Methods:**

Fellow eyes of 34 cases with unilateral PEX were evaluated by slit-lamp and gonioscopy. Findings associated with PEX were recorded.

**Results:**

Mean age was 67.8±8.1 years (range 55-86 years). Twenty-five patients (73.5%) had pigmentation in the inferior angle and 23 patients (67.6%) had Sampaolesi’s line located on the inferior angle in fellow eyes. The other most common findings were loss of peripupillary ruff in 10 patients (29.4%) and pigment dispersion following pupil dilation in 14 patients (41.1%).

**Conclusion:**

Pigmentation in the inferior angle and Sampaolesi’s line on the inferior angle seem to be the most common early findings associated with PEX. Special attention should be paid to these findings in cases with ocular hypertension for proper management.

## INTRODUCTION

In pseudoexfoliation (PEX) syndrome, extracellular fibrillary material is deposited throughout the anterior segment, particularly over the anterior lens capsule in a characteristic double concentric ring pattern with a clear zone between the rings.^1,2^ Clinically, ocular involvement in PEX syndrome is described as unilateral in half of the patients.^3,4^ In an electron microscopic study, Parekh et al.^5^ reported that 26 of 32 patients (81%) with clinically unilateral PEX had PEX material on either the lens capsule or conjunctival samples of the clinically unaffected eyes. Furthermore, several reports on the follow-up of patients with unilateral PEX documented that a proportion of the unilateral cases became bilateral.^6,7^ This suggests that unilateral PEX is in fact a bilateral but asymmetric condition.

In this study we aimed to determine the early signs of PEX syndrome in fellow eyes of cases with unilateral PEX syndrome.

## MATERIALS AND METHODS

The study comprised 68 eyes of 34 patients aged 67.8±8.1 (range 55-86) years with unilateral PEX syndrome who were examined between January 2014 and March 2015. Written informed consent was obtained from all patients enrolled in this cross-sectional non-interventional study. The study was approved by the Local Ethics Committee and conducted in accordance with the principles of the Declaration of Helsinki. None of the patients had undergone ocular surgery or laser treatment. Patients with any ocular disease (other than glaucoma) which might interfere with gonioscopy and fundus examination results, such as corneal opacities, cataracts, or retinal lesions; the presence or history of ischaemic, compressive, or inflammatory optic neuropathies; refractive errors greater than ±6 diopter (D); or inflammation or trauma in any eye, were excluded. Unilateral exfoliation was defined clinically as the presence of biomicroscopically detectable exfoliation material on the anterior lens capsule or at the pupillary border in one eye after pupillary dilatation with 10% phenylephrine hydrochloride. Eyes were classified as clinically normal if there was no evidence of exfoliation material on the pupil, lens or angle.

All participants underwent a detailed ophthalmologic examination including slit-lamp examination, intraocular pressure (IOP) measurement using Goldmann applanation tonometry, gonioscopy and +90 D fundus examination. Gonioscopy, employing the Goldmann tri-mirror goniolens under standard conditions, was performed to visualize the angle and the angle recess; trabecular pigmentation was noted and open angle and angle closure suspects were defined according to the International Society for Geographical and Epidemiological Ophthalmology classification.^8^

IOP measurements were performed between 08:00 AM and 10.00 AM. The highest IOP obtained from the medical history was accepted as “maximum IOP”.

Optic disc assessments were performed biomicroscopically with a 90-D lens and the average of vertical cupping was recorded as the cup-to-disc ratio.

### Statistical Analysis

Statistical analysis was performed using SPSS software version 10. Differences between eyes were analyzed using Student’s t-test and Mann-Whitney U test for non-parametric variables, while the chi-square test was used for analysis of differences among proportions. Statistical significance was set at 0.05.

## RESULTS

The records of 68 eyes of 34 patients with unilateral PEX syndrome were evaluated. Mean patient age was 67.8±8.1 (range 55-86) years, and the male:female ratio was 20/14.

The mean IOP was 22.35±7.33 mmHg in eyes with PEX and 17.0±2.17 mmHg in normal eyes (p=0.001). The maximum IOP was 29.13±9.6 mmHg in eyes with PEX and 18.7±2.7 mmHg in normal eyes (p<0.001).

Topical anti-glaucomatous medication was being applied in 25 eyes with PEX and in 17 normal eyes (p=0.045). The disc cupping ratio was higher in eyes with PEX (0.58±0.25) than in normal eyes (0.17±0.07) (p=0.033).

Twenty-five eyes (73.5%) had pigmentation in the inferior angle and 23 eyes (67.6%) exhibited Sampaolesi’s line located on the inferior angle. The other most common findings were loss of peripupillary ruff in 10 eyes (29.4%) and pigment dispersion following pupil dilation in 14 eyes (41.1%). The findings are shown in Tables 1, 2, 3 and 4.

## DISCUSSION

There has been great variability in the prevalence of PEX syndrome, from 0.5% up to 33%.^1,9^ The prevalence of PEX syndrome in Turkey was reported as ranging between 11.2% and 17.7% in different studies.^10,11,12^

PEX syndrome is a systemic disease which leads to the development of glaucoma in up to 50% of cases.^13,14,15^ Additionally, PEX syndrome is the most common identifiable cause of open-angle glaucoma, and accounts for an estimated 25% of the open-angle glaucoma worldwide.^16^ The pathogenesis of exfoliation glaucoma represents an imbalance between aqueous humour secretion, outflow facility and optic nerve microcirculation.^17^ Blockage of the trabecular meshwork by pigment and PEX material, and trabecular cell dysfunction eventually result in elevated IOP, which leads to PEX glaucoma.^16^

PEX syndrome is basically bilateral with asymmetric clinical manifestations,^18^ related with the rate of production, aggregation, and accumulation of the abnormal extracellular material in each eye.^19^ Immunohistochemical and electronmicroscopic studies in autopsy eyes obtained from donors with clinically unilateral PEX have also revealed that exfoliation is actually asymmetric rather than truly monocular.^18,20^ PEX material has been demonstrated on the iris and ciliary epithelia and in the dilator muscle of the iris in fellow eyes of clinically unilateral donors,^19^ and vasculopathy in iris vessels has been reported to precede the appearance of exfoliative material in the posterior and anterior chambers of the eye.^20^ The vasculopathy and the consequent iris hypoperfusion have been documented in both glaucomatous and non-glaucomatous eyes with PEX and therefore, to some extent, are independent of IOP.^21^

In this study, we accepted that the PEX is a bilateral but asymmetric disease and investigated early clinical findings in the eye that seems to be “normal”. This is the first study which investigated anterior segment and iridocorneal angle in this context in detail. In our study, the most common signs in fellow eyes were pigmentation in the inferior angle (73.5%) and Sampaolesi’s line located on the inferior angle (67.6%). The other common findings were loss of peripupillary ruff (29.4%), and pigment dispersion following pupil dilation (41.1%).

Rao^22^ conducted a study to compare clinical findings and retinal nerve fiber layer (RNFL) thickness in unilateral and bilateral PEX cases in order to identify predictors of early glaucomatous damage on optical coherence tomography. From a total of 32 unilateral PEX cases, 7 subjects demonstrated RNFL thinning in the clinically normal fellow eye; all of these eyes had evidence of pupillary ruff atrophy on slit-lamp examination in the absence of evident exfoliation material in the eye. Similar ruff atrophy with RNFL thinning was detected in 38 of 59 bilateral and in 16 of 32 unilateral cases. The authors suggested that iris sphincter abnormality, clinically detected as pupillary ruff atrophy, may reflect early glaucomatous damage. Loss of peripupillary ruff (29.4%) was one of the common findings in our study as well. We know already that eyes with PEX are under greater risk for ocular ischaemic conditions because of pathological vascular alterations associated with PEX.^7^ Vasculopathy in iris vessels leads to iris hypoperfusion and precedes clinical visualization of the PEX material.^23^

Omura et al.^24^ compared ocular parameters between PEX-positive and PEX-negative eyes in 49 subjects with unilateral PEX syndrome and reported that, compared to PEX-negative eyes, PEX-positive eyes had lower visual acuity, higher IOP, lower corneal endothelial cell density, thicker lenses, lower anterior chamber volume, higher flare values and required more antiglaucoma medications. The refractive errors, central corneal thickness and anterior chamber depth did not differ between the two groups. In that study, values such as corneal endothelial cell density, lens thickness, and anterior chamber volume were investigated. However, in our study we especially focused on studying the iridocorneal angle in more detail.

## CONCLUSION

This study presents a thorough investigation of anterior segment and iridocorneal angle changes in patients with unilateral PEX syndrome. Pigmentation and Sampaolesi’s line at the inferior angle seem to be the earliest findings associated with PEX. Special attention should be paid to these findings in cases with ocular hypertension for proper management.

### Ethics

Ethics Committee Approval: It was approved by the Ethics Committee of Başkent University Clinical Research (no: 15-20), Informed Consent: Obtained.

Peer-review: Externally peer-reviewed.

## Figures and Tables

**Table 1:**
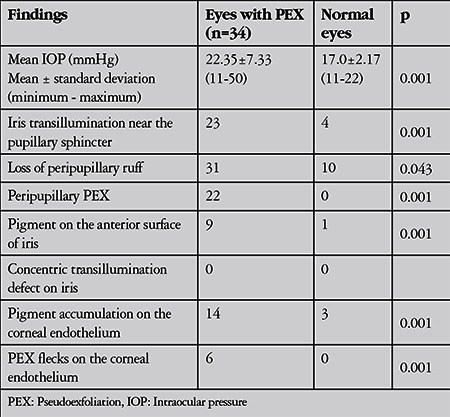
Comparison of anterior segment findings between eyes with pseudoexfoliation and normal fellow eyes

**Table 2:**
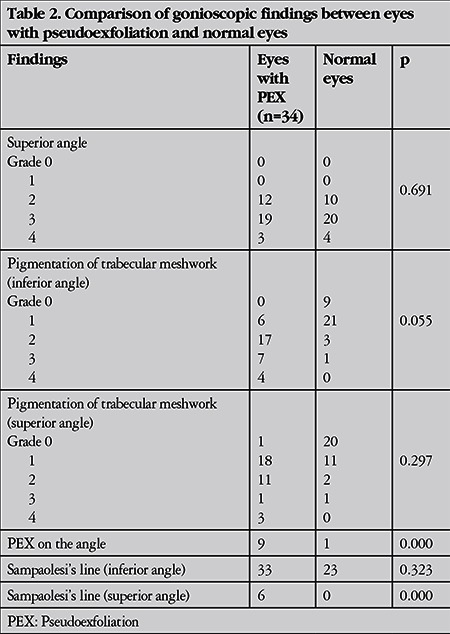
Comparison of gonioscopic findings between eyes with pseudoexfoliation and normal eyes

**Table 3:**
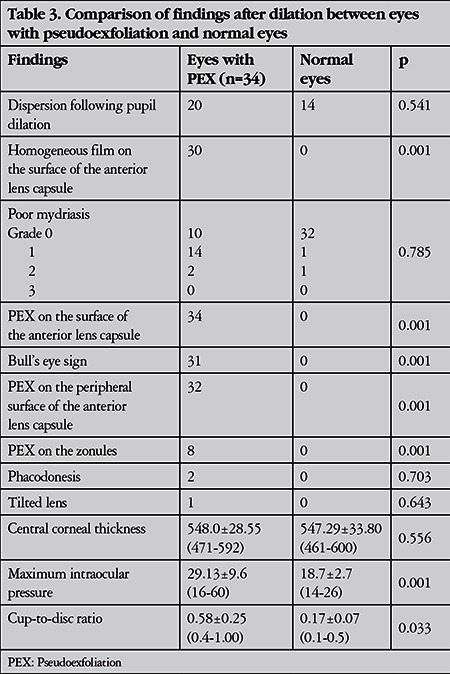
Comparison of findings after dilation between eyes with pseudoexfoliation and normal eyes

**Table 4:**
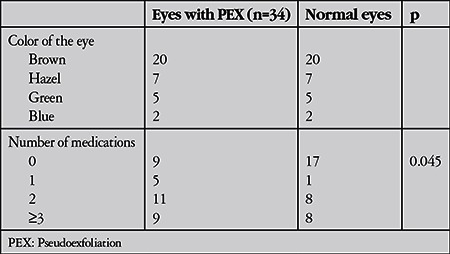
The comparison of eye colors and number of medications between eyes with pseudoexfoliation and normal eyes
